# Heterotypic Multicellular Spheroids as Experimental and Preclinical Models of Sprouting Angiogenesis

**DOI:** 10.3390/biology11010018

**Published:** 2021-12-23

**Authors:** Igor V. Vakhrushev, Elizaveta K. Nezhurina, Pavel A. Karalkin, Anastasia V. Tsvetkova, Nataliya S. Sergeeva, Alexander G. Majouga, Konstantin N. Yarygin

**Affiliations:** 1 Laboratory of Cell Biology, Institute of Biomedical Chemistry, 119121 Moscow, Russia; seagleam@gmail.com; 2 P.A. Hertsen Moscow Oncology Research Center, National Medical Research Radiological Center, 125284 Moscow, Russia; eliznezhurina@gmail.com; 3 Institute for Cluster Oncology, Sechenov University, 119435 Moscow, Russia; pkaralkin@gmail.com; 4 Department of Biology, Pirogov Russian National Research Medical University, 117997 Moscow, Russia; prognoz.01@mail.ru; 5 Faculty of Chemical and Pharmaceutical Technologies and Biomedical Products, D. Mendeleev University of Chemical Technology of Russia, 125047 Moscow, Russia; alexander.majouga@gmail.com

**Keywords:** tissue spheroids, sprouting angiogenesis, endothelial cells, perivascular cells, mesenchymal stem cells, in vitro angiogenesis models

## Abstract

**Simple Summary:**

In adult mammals, including humans, new blood vessels are formed mostly via a process named sprouting angiogenesis. In physiological conditions, angiogenesis can improve blood perfusion and oxygen supply of organs and tissues, but it can also be harmful. For example, active angiogenesis promotes tumor growth. To be able to regulate angiogenesis, it is necessary to understand the underlying molecular and cellular mechanisms, and this, in turn, requires the development of suitable models. Here we review the existing models of sprouting angiogenesis and describe an in vitro approach that is suitable for further deciphering its cellular and molecular mechanisms, preclinical drug testing, and research in regenerative medicine under conditions close to the in vivo conditions. This approach is based on the use of 3D tissue aggregates named spheroids and consisting of endothelial cells lining the inner surface of blood vessels, one or more other types of cells forming the vessel wall, and the extracellular matrix. It has a great potential for further refinement for use in such applications as formation of prevascularized tissues for bioprinting and tissue engineering.

**Abstract:**

Sprouting angiogenesis is the common response of live tissues to physiological and pathological angiogenic stimuli. Its accurate evaluation is of utmost importance for basic research and practical medicine and pharmacology and requires adequate experimental models. A variety of assays for angiogenesis were developed, none of them perfect. In vitro approaches are generally less physiologically relevant due to the omission of essential components regulating the process. However, only in vitro models can be entirely non-xenogeneic. The limitations of the in vitro angiogenesis assays can be partially overcome using 3D models mimicking tissue O_2_ and nutrient gradients, the influence of the extracellular matrix (ECM), and enabling cell-cell interactions. Here we present a review of the existing models of sprouting angiogenesis that are based on the use of endothelial cells (ECs) co-cultured with perivascular or other stromal cells. This approach provides an excellent in vitro platform for further decoding of the cellular and molecular mechanisms of sprouting angiogenesis under conditions close to the in vivo conditions, as well as for preclinical drug testing and preclinical research in tissue engineering and regenerative medicine.

## 1. Introduction

Development of the experimental models for tissue vascularization research was substantially accelerated in early 1960s after Folkman et al. [1,2] demonstrated that the growth of a tumor depends on how well it is vascularized. The earliest models for evaluation of the mechanisms of blood vessel growth included the in vivo chicken chorioallantoic membrane model and the ex vivo model utilizing hydrogel-embedded aortic ring explants [3,4,5]. The development of the protocols of ECs isolation and long-term culture in the early 1970s provided an opportunity to establish the first in vitro models [6,7]. From 2D tube formation assays to 3D microfluidics, in vitro models have been actively improved seeking to better mimic the processes of blood vessels formation taking place in vivo [8].

In mammals, small blood vessels are formed by two processes, vasculogenesis and angiogenesis. Vasculogenesis is the de novo generation of a primitive vascular network via mesoderm-derived endothelial precursors (angioblasts) migration, differentiation, and alignment into vascular tubes (reviewed in [9]). This process primarily occurs during embryonic development. After birth, the blood vessels propagate via the process named angiogenesis (for review see [10]). Angiogenesis occurs under physiological stress, in the course of recovery from injuries, and in vessel growth-inducing pathological conditions such as cancer or ischemia. New blood vessels mainly form by sprouting from the pre-existing ones, as briefly described below. In response to angiogenic signals, some ECs start secreting matrix metalloproteinases (MMPs), dissolve the basal membrane, become motile, extend filopodia, and invade the ECM, turning into the so-called tip cells. Following the tip cells, other ECs called stalk cells proliferate to support sprout elongation and establish lumen formation. Tip cells anastomose with cells from neighboring sprouts to build vessel loops. The initiation of blood flow, generation of the basement membrane, and the recruitment of mural cells stabilize the newly formed net of small blood vessels. The sprouting process reiterates until proangiogenic signals abate and quiescence is reestablished. The angiogenic sprouting is described in more detail in the next section. In view of the above, the in vitro models of blood vessel formation can be classified into two main categories—vasculogenesis and angiogenesis models. The currently used angiogenesis models are based on the sprouting of ECs into a hydrogel matrix from a monolayer or 3D culture (both mimicking the blood vessel wall). There are three types of in vitro models of angiogenesis assessment: invasion assay that was developed by the Davis and Bayless group [11,12,13], fibrin bead assay by Nehls et al. [14], and spheroid sprouting assay by Korff et al. [15,16]. The latter is based on the use of tissue spheroids as a 3D culture system and has the potential to outperform the other two because, conceivably, it can imitate more substantial features of the in vivo microenvironment of micro-vessels. Moreover, the possibility to co-culture different types of cells and the ability of spheroids to fuse forming 3D tissue constructs makes heterotypic spheroids a promising tool at the cutting edge of tissue engineering, enabling creation of complex tissue equivalents, with a pre-formed vascular system.

Here we present a review of the existing models of sprouting angiogenesis that are based on the use of heterotypic spheroids comprising of Ecs and other types of cells. We maintain that this approach provides an excellent in vitro platform for further deciphering of the cellular and molecular mechanisms of sprouting angiogenesis under conditions that are close to the in vivo conditions, as well as for preclinical drug testing and preclinical research in tissue engineering and regenerative medicine.

## 2. Sprouting Angiogenesis

Angiogenesis is the process by which new blood vessels are formed from the existing blood vessels either through “sprouting” of Ecs or through “intussusception” (vascular splitting) [17,18]. Angiogenesis should be distinguished from vasculogenesis, which is the process of de novo blood vessel formation during embryonic development. Though it has been shown that intussusceptive angiogenesis takes place in embryogenesis, during the postnatal vasculature remodeling, and in pathological conditions, and that both angiogenic mechanisms can co-exist in certain physiological and pathophysiological settings [19], angiogenic sprouting is probably the prevailing and most common mechanism of angiogenesis. 

**Figure 1:**
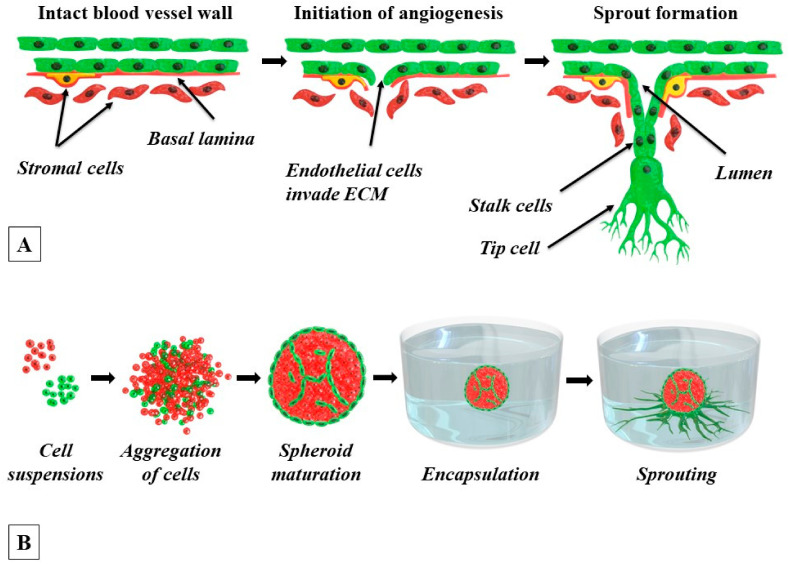
Schematic diagram of natural angiogenesis and it’s modeling in vitro using tissue spheroids. (**A**) The key steps of in vivo angiogenesis. Some ECs (green) from the vascular wall are activated in response to signals from the surrounding tissues. They initiate the cascade of processes, such as secretion of MMPs, decomposition of the basal lamina, migration towards the source of chemotactic stimuli, proliferation, and tube formation, providing the sprouting of cells from the mature endothelial layer of the vessel wall. The tip cell produces pseudopodia that guide the development of the capillary sprout as it grows into the surrounding tissue. The stalk cells provide the elongation of the sprout through extensive proliferation. As the sprouting progresses, the surrounding stromal cells (pericytes (yellow), fibroblasts (red), and also the mesenchymal stem cells, MSCs) begin to attach to the growing sprout, thus providing support and stabilization. At the late stages, the capillary sprout hollows out to form a tube. (**B**) Workflow steps of 3D angiogenesis sprouting assay. Isolation and in vitro expansion of ECs (green color) and stromal (red color) cells; mixing several populations of single-cell suspensions in different ratios and transferring to the low-adhesion culture plates or molds; production of hybrid tissue spheroids with differential localization and distribution of ECs and stromal cells (3–4 days); transferring of mature spheroids into the hydrogel with angiogenic factors; induced formation of angiogenic sprouts (2–3 days); now the in vitro assay is ready for subsequent study of molecular mechanisms or drug discovery.

The process of sprouting is triggered when Ecs are activated by the pro-angiogenic paracrine signals that are coming from their tissue microenvironment in response to a growing need from tissue parenchymal cells for oxygen and nutrients or to pro-angiogenic factors that are secreted by injured cells or tumor cells. The list of the most potent and physiologically relevant inducers of sprouting angiogenesis includes vascular endothelial growth factors, fibroblast growth factors, hypoxia-inducible factors, and angiopoietins [20]. In vivo, in response to the angiogenic stimuli, endothelial progenitors that are present in the pre-existing vessels are activated, express metalloproteases, dissolve the basal membrane, invade the surrounding matrix, start to proliferate, and form the sprouting vessel bud [21]. The latter comprises two types of Ecs: the tip cells and the stalk cells. The tip cells are characterized by the ability to dissolve the ECM, migrate, and lead the nascent sprout towards the source of the angiogenic stimuli. The highly proliferative stalk cells follow the tip cell, provide sprout elongation, and form the trunk of the newly formed capillary. Pericytes that are adjacent to endothelial cells through the vessel basement membrane also play an important role in angiogenesis [22]. Upon angiogenic activation, they secrete MMPs, detach from the vessel wall by proteolytic degradation of the basement membrane, and support angiogenesis by remodeling the ECM and by stabilization of the growing sprout [20]. The initial stages of sprouting angiogenesis are schematically presented in Figure 1A.

Lumen formation (tubulogenesis) is another critical step in vascular development [13,23,24]. It is a vascular endothelial growth factor (VEGF)-A-driven process. There are two different ways of lumen formation that have been proposed more than a century ago: cord hollowing by Billroth [25] and cell hollowing by Sabin [26]. Later, it was shown that both cord and cell hollowing are common mechanisms of vascular lumen formation and are in place in different tissues across the animal phylogenetic tree [27]. Cord hollowing includes cytoskeletal changes that induce the neighboring apical stalk cell surfaces to separate from each other to form fluid-filled cavities between the cells. At the same time, the ECs forming the sprout and their nuclei synchronously elongate and flatten. Later the formed cavities merge to form the vessel lumen. Alternatively, according to the cell hollowing hypothesis, the endothelial stalk cells form large vacuoles through pinocytosis [23,28]. The vacuoles of the adjacent stalk cells eventually fuse with plasma membranes and coalesce into a hollow lumen. 

Fibroblasts and pericytes contribute to tube formation in the angiogenic sprout by releasing the tubule formation-stimulating factors (VEGF, FGF-2, IL-3, SDF-1a, and others) and components of ECM [29,30]. The sprouts grow towards the source of VEGF or other angiogenic stimulus. When two sprouts with lumens meet, they merge and form a new continuous capillary loop [31]. 

This list of the main stages of sprouting angiogenesis at the cellular level, and much more that is known about it, comes from studies that have been performed using various in vivo and in vitro models. Numerous model systems for studying angiogenesis have been developed but still there is no “gold standard” assay for studying this process and testing the substances impacting it [32]. In vitro angiogenesis models, such as proliferation, migration, and tube formation assays, are characterized by high precision and provide control of many parameters of the angiogenic process. However, most in vitro assays are still performed in 2D cultures and many of them utilize endothelial cells only and do not involve other types of cells participating in angiogenesis, such as pericytes, fibroblasts, and myocytes. The more holistic nature of the in vivo models provides additional biological and clinical relevance, but in vivo experiments do not always allow adequate control of their parameters. Besides, in vitro models can be based exclusively on human cells. Accordingly, there is an urgent need to develop and employ more sophisticated in vitro models of angiogenesis allowing the better reproduction of the conditions existing in vivo.

## 3. Endothelial and Perivascular Cell Types 

Obviously, ECs are the main type of vascular cells, without which blood vessels cannot exist. Accordingly, they are an essential element of any in vitro angiogenesis model. In the early 1970s, the first protocols of long-term culture of human umbilical cord vein ECs (HUVECs) were established and gave rise to the development of several in vitro angiogenesis models [6,7]. Although since then, the list of the protocols of different tissue-specific EC types isolation has been greatly expanded, HUVECs remain one of the most popular EC types, because they can be easily isolated and cultured and have an already fully differentiated endothelial phenotype, including the expression of CD31 (platelet endothelial cell adhesion molecule, PECAM), CD144 (VE-cadherin), and Factor VIII antigen (von Willebrand Factor) [33]. However, ECs that are isolated from microvessels, as well as from arteries and veins of different caliber supplying various tissues with blood have been also isolated, cultured, and used in angiogenesis research, as were ECs that were taken from donors with cancer, diabetes type I or type II, eye pathologies, and other diseases [34,35,36,37,38]. 

Currently, there is a growing interest in the blood-derived EC populations with varying properties, such as umbilical cord blood-derived outgrowth endothelial cells (OECs), endothelial progenitor cells (EPCs), and umbilical cord blood-derived endothelial colony forming cells (ECFCs). OECs are isolated from the mononuclear fraction of blood by plating mononuclear cells in the EC-supporting culture medium and selecting the population of late OECs which appear after two to three weeks of culture, demonstrating specific morphology and shorter doubling time compared to early OECs (which appear within one week of culture) [39,40]. EPCs were first described and isolated by Asahara et al. in 1997 [41]. These bone marrow-derived cells circulate in peripheral blood and are positive for both human hematopoietic stem cells markers CD34 and CD133 and for endothelial markers, such as VEGF receptor 2 (VEGFR-2) [42]. It is known that they are present in high concentration in the umbilical cord blood. ECFCs constitute a subgroup of EPCs with a high proliferative and clonal capacity, although no ECFC-specific marker has been identified yet [43,44].

Perivascular or mural cells of the small blood vessels that are located on the outer side of the basal membrane are closely associated with the endothelial layer, both structurally and functionally. Depending on the blood vessel type, they can be divided into two main categories—vascular smooth muscle cells (VSMCs) constituting the major cell type in the media layer of arterioles [45], and pericytes that are present in the capillaries and post-capillary venules [46]. VSMCs and pericytes take part in the regulation of the vessel wall permeability and tone. They are essential for maintaining small vessel structural integrity. There is no single marker or group of markers that is absolutely specific for perivascular cells as they share markers with several cell types. The commonly accepted combination of markers that are used for perivascular cells identification includes nerve/glial antigen 2 (NG2), CD146, α-smooth muscle actin (α-SMA), and platelet-derived growth factor receptor beta (PDGFR-β) [20]. It is known that MSCs of different origins are capable of transforming growth factor beta (TGF-β) signaling-regulated differentiation into mural cells [47,48]. 

MSCs, a heterogenous stromal cell population that was first described by the Friedenstein’s group [49] and was later named by Caplan [50], and are now designated by different names, such as mesenchymal stromal cells or mesenchymal stromal stem-like cells, are known to easily engage in crosstalk with many other cell types and are definitely involved in angiogenesis in several ways. MSCs are isolated from tissue samples that are disintegrated to single cells by enzymatic digestion on the basis of their ability to adhere to cultural plastic. It is known that MSCs of different origins participate in the paracrine regulation of angiogenesis in tumors [51] and other pathological conditions [52]. MSCs also take part in the stabilization of growing microvessels directly [52]. As mentioned above, in vitro they are capable of TGF-β signaling-regulated differentiation into mural cells [47,48]. Perivascular cells and MSCs have very similar expression profiles of surface markers and both can adhere to plastic [53]. Accordingly, pericytes and probably all mural cells are frequently co-isolated with other mesenchymal cells and constitute subpopulations of MSCs which, in culture, probably gradually lose their identity that was initially determined by their perivascular position in vivo [54]. With regard to the above, not only pericytes and VSMCs, but MSCs as well, are candidates for the role of co-culture partners of ECs in heterotypic spheroid-based angiogenesis studies.

## 4. 3D Tissue Spheroids Comprised of ECs and Perivascular Cells

Tissue spheroids are cell aggregates that are formed in non-adhesive conditions, preventing the attachment of cells to the bottom of a culture flask or other substrates that are used to maintain the 2D cultures. They can be generated by various methods, including spontaneous spheroid formation in the ultra-low binding plates, spontaneous in the ultra-low binding plates containing hydrogels, “hanging drop” technique, spheroid formation in suspension cultures in bioreactors, or using magnetic levitation [55]. Here, we shall concentrate on the first two, since they are up-to-date, informative, and cost-effective.

The self-assembly of spheroids in a cell suspension is presented in Figure 1B. In the absence of an adhesive substrate, the cells begin to aggregate with neighboring cells by cadherins, a subclass of cell adhesion molecules (CAMs) [55]. The presence in the microenvironment of fibrillar proteins that are rich in the RGD, PHSRN, GFOGER, RGDWXE, and other motifs in their primary structure, such as the ECM proteins fibronectin, laminin, vitronectin, fibrin, and collagen, facilitates spheroid initiation and further outbound migration of cells from the formed spheroids [55,56,57]. Some of these proteins provide better results after partial denaturation. The integrin-fibrillary protein interactions contribute to the physical convergence of the cells to form compact aggregates and induce the upregulation of the expression of cadherins and their assembling to form clusters on the surface of cells. Next, the cadherin-cadherin interactions between neighboring cells tighten the cell-cell connections further and promote additional spheroid compaction. The tissue spheroids sharing many common biological features with a number of normal or diseased tissues, such as vascularized tumors, blood-brain barrier, and cardiac tissue, were produced [58,59,60]. The ability to co-culture two or more cell types makes 3D tissue spheroids a promising tool to model heterotypic cell-cell interactions and brings this 3D system to the cutting edge of tissue engineering and drug screening [55,56,61,62].

Since Korff and Augustin introduced the EC-spheroid model in 1998 [63] and later a collagen-gel-based 3D angiogenesis model that was based on EC-spheroids that are embedded in a collagen gel [15], much attention has been directed to the application of tissue spheroids in angiogenesis modeling. In vivo, the growth of new vessels is regulated by the perivascular niche and involves the activation and recruitment of perivascular cells and ECM modulation. Accordingly, realistic angiogenesis models should include perivascular cells and ECM elements. In monoculture, the ECs do not tend to form 3D structures, probably because in their natural environment in blood vessels they are organized into one-cell-thick tubes. Therefore, the protocols of EC monoculture spheroids formation include the addition of methylcellulose as a suspending agent that does not allow spheroids to sediment [35,64]. In contrast to ECs, stromal cells easily self-organize into 3D multicellular aggregates, and, accordingly, the presence of perivascular stromal cells improves the spheroid formation process [65]. 

**Figure 2:**
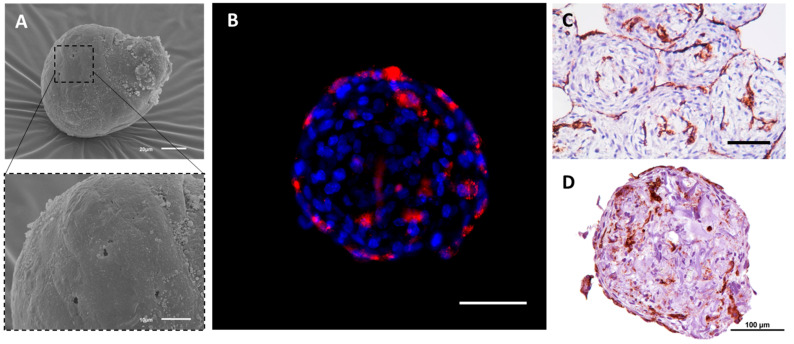
The structure and internal organization of heterotypic tissue spheroids that are assembled of HUVECs and human umbilical cord MSCs (UCMSCs). The formation of the spheroids: the suspension of cells (100 µL per well, 1000 cells per spheroid, 1:1 ratio) was added to the non-adhesive U-bottom 96-well plate (Corning, Corning, NY, USA). After 72 h, the spheroids were collected and studied using scanning electron microscopy (SEM), immunofluorescence (IF), and immunohistochemistry (IHC). (**A**) SEM of the heterotypic HUVEC-UCMSC spheroids at day three in culture. The scale bars correspond to 20 μm (top) and 10 μm (bottom). (**B**) IF study of the formation of the 3D inner endothelial structures inside HUVEC-UCMSC spheroids. HUVECs were labeled with PKH26 (red, Sigma, USA) prior to tissue spheroids formation. The mixed tissue spheroids were incubated with DAPI (1:1000, Invitrogen, Waltham, MA, USA) to counterstain cell nuclei (blue). The scale bar corresponds to 100 µm. (**C**,**D**) IHC staining of spheroids for CD31, a marker of HUVECs. Prior to histological slides preparation, 20 spheroids were collected and placed into one well of the non-adhesive U-bottom plate for two hours to ensure their fusion. The entrapped in molten agarose tissue spheroids were fixed in 10% buffered formalin (pH 7.4) for 24 h and embedded in paraffin (Biovitrum, St Petersburg, Russia). 5 µm thick sections were cut with Microtome HMS 740 (Thermo Fisher Scientific, Waltham, MA, USA) and mounted on poly-L-lysine coated glass slides. Primary polyclonal rabbit antibodies to human CD31 (PECAM) were used in 1:100 dilutions. The nuclei were counterstained with Mayer’s hematoxylin. Finally, the sections were dehydrated and enclosed in Bio-Mount (Bio Optica, Milano SPA, Italy). The scale bar corresponds to 100 µm.

Importantly, it was shown that ECs that are co-cultured with adhesive perivascular cells such as VSMCs, pericytes, fibroblasts, MSCs, and osteoblasts in heterotypic tissue spheroids, demonstrate a very specific localization pattern where they form a monolayer at the spheroid surface and a primitive 3D capillary bed-like network within the spheroid core [66,67,68] (Figure 1B and Figure 2 show our own unpublished data [69]). The mechanism of such ECs spatial distribution is not fully understood and needs further investigation. According to Steinberg’s differential adhesion hypothesis, the aggregation of two and more different cell types in tissue spheroids promotes specific spatial cell localization patterns (a phenomenon called “cell sorting”) that affects the spheroid properties [70,71]. Different adhesive cell types demonstrate different cell sorting behaviors, and the mechanisms of cell sorting patterns look controversial [71,72]. With regard to the endothelial-perivascular spheroids, it is known that the formation of a vascular network within the tissue spheroids depends primarily on the diameter of the spheroid determining the presence of a necrotic zone. Tissue spheroids have diffusion limitations of 150–200 µm that are applicable to many molecules, including O_2_ [55]. Thus, tissue spheroids with a diameter above 500 µm always have a necrotic core in the center that is surrounded by a quiescent viable cell zone and a peripheral layer of proliferating cells [55]. Eckermann et al. [66] clearly demonstrated that the formation of a necrotic zone in the ECs-fibroblasts mixed spheroids exceeding 650 µm in diameter leads to massive cell death due to apoptosis and prevents the formation of ECs vascular network. In the majority of studies, the ECs/perivascular cells ratio of 1:1 is considered as an optimal ratio [73,74,75], however in a rather early study it was shown that tissue spheroids that contained up to 10 % of ECs developed dense endothelial networks, while the use of higher percentages of ECs led to less elongated structures that were similar to cell clumps [66]. Most probably, the optimum ratio should be determined in each particular case. Indeed, good quality spheroids could be framed using ECs/MSCs ratios between 1/3 and 5/1 [34,76,77]. As part of our own work, we have successfully generated heterotypic spheroids, each containing about 1000 cells and consisting of HUVECs and umbilical cord MSCs at a 1:1 ratio (unpublished data) [69]. The spheroids were characterized by means of scanning electron microscopy (Figure 2A) and confocal microscopy (Figure 2B). The distinctive distribution of both cell types within the spheroids has been observed (Figure 2B,D). Amongst the broad functional testing of the obtained spheroids, their capacity to fuse has been demonstrated indicating good viability and general condition of the cells comprising them (Figure 2C).

Marshall et al. [73] identified regulatory pathways that were involved in the spatial organization and functioning of heterotypic spheroids incorporating ECs and MSCs. In this study, the cells were pre-treated with the inhibitors and antagonists of key signaling pathways that were associated with EC migration and behavior, including the inhibitors of integrin-linked kinase, Notch pathway, and antagonists of PDGFR, epidermal growth factor receptor (EGFR), and fibroblast growth factor receptor (FGFR). Blocking of the integrin-linked kinase and PDGFR resulted in the formation of a more prominent EC network. The inhibition of the Notch signaling promoted shifting of the EC capillary-like structures to the periphery of spheroids, while the blocking of FGFR led to a disruption of the EC network formation and induced ECs aggregation within the center of the spheroid. The blocking of EGFR appeared to have no effect on EC network formation. These data demonstrate that ECs behavior in heterotypic spheroids in the presence of MSCs is influenced by a number of endogenous signaling systems, especially by the PDGF signaling pathway.

Several studies demonstrated the presence of lumens within some of the endothelial cords constituting the internal endothelial network within the heterotypic spheroids [65,68]. It was shown that lumen formation in 3D spheroids is regulated by the mechanisms similar to those active in vivo. Sonic Hedgehog morphogen, being expressed in the process of endothelial tube formation during neovascularization after trauma [78] and during wound healing [79], also promotes lumen formation in tissue spheroids. It regulates the expression of angiogenic genes, in particular encoding for cytoskeleton proteins and proteins that are related to pseudopodia-associated cell migration that is crucial for adequate lumen formation and guidance of the tip cells [68]. 

Perivascular and other stromal cells not only assist 3D endothelial network formation, but influence ECs viability and are engaged in ECM deposition. The presence of perivascular cells in heterotypic spheroids prevents ECs apoptosis and prolongs their lifespan during long-term culture in comparison to EC monoculture spheroids [65]. Spheroids comprising of VSMCs or fibroblasts improve ECs viability under low serum conditions (2% fetal calf serum, FCS) [16,80]. The transmission electronic microscopy showed that ECs in co-culture spheroids established more junctional complexes than in EC monoculture spheroids, and also some microvesicular bodies and extracellular vesicles were detected, suggesting functional cell-cell interactions [16,80,81]. In 10 day-old spheroids, some ECs formed intracellular vacuoles and EC cords that contained lumens and basement membrane, indicating the self-assembly of real microvessels [65]. It was also shown that the endothelial expression of PDGF was completely down-regulated in mixed EC-VSMC spheroids over time [16], while the OEC-MSC spheroids accumulated vast amounts of fibronectin and collagen type IV-proteins that are specific for the basal membrane of capillaries [81]. In EC-osteoblast co-culture, spheroids osteocalcin and alkaline phosphatase as well as VEGF were detected suggesting the tissue-specific origin of the perivascular cells, in this particular case probably originating from osteoblasts [82]. It was also shown that ECs that were cocultured with VSMCs in mixed spheroids expressed N-cadherin that is known to be a marker of EC-pericyte communication during angiogenesis [83,84].

## 5. The Functionality of Prevascularized 3D Tissue Spheroids

The concept of prevascularized spheroids preparation for tissue engineering applications has been gaining increasing attention during the last decade [65,85,86,87]. In this regard, it was very important to evaluate the ability of the primitive microvascular network of prevascularized spheroids to merge with the host’s vascular system. Several research groups have reported the in vivo functionality of prevascularized tissue spheroids made up of human cells after subcutaneous implantation into the immuno-compromised mice [68,88,89]. The presence of erythrocytes and lectin in the lumens that were lined with human CD31+ cells revealed after the injection of fluorescent lectin into the tail vein clearly proved the perfusion of the implant. Moreover, the vascular structures within spheroids anastomosed with the host vasculature. It is worth mentioning that cord-like non-lumenized structures that were present in vitro were not observed after implantation, which can be attributed to their regression while lumenized structures were probably maintained by the blood flow [68]. 

Chang et al. [75] used human EC monoculture spheroids and heterotypic human EC-pericyte spheroids to assess the role of pericytes in maintaining spheroid functionality in vitro and in vivo. In vitro human pericytes that were separated from EC monoculture spheroids by a semipermeable membrane in the Transwell setting, or the addition of the pericyte-conditioned medium increased ECs sprouting via a hepatocyte growth factor (HGF)-dependent mechanism. Endothelial sprouts that were formed in the EC–pericyte spheroids became promptly inoculated by pericytes. The blockade of PDGF signaling inhibited inoculation and enhanced sprouting, suggesting the regulatory role of pericytes in the formation of the microvascular net. After four weeks following the implantation of endothelial monoculture spheroids or EC–pericyte coculture spheroids that were embedded in collagen gels into immunodeficient mice human, EC-lined perfused microvessels containing mouse erythrocytes were observed [75].

## 6. 3D Spheroid Sprouting Model

The 3D spheroid sprouting assay was first acknowledged in 1998 as an in vitro angiogenesis model that was based on 3D ECs monoculture spheroid that was embedded in collagen or fibrin to induce sprouting, i.e., the formation of tubular capillary structures [63]. Later, this assay was modified and switched to the use of heterotypic EC–perivascular cell spheroids to better mimic the microenvironment of the vascular niche [48,75]. This latter version allowed modeling of both endothelial-perivascular cell interactions during the formation of sprouts and the role of ECM in this process. The process of tissue spheroids preparation was described in Section 4 in this paper. The procedure of the application of the tissue spheroid technology to angiogenesis modeling and studies of the influence of various factors on this process are presented below. The combination of both is schematically represented in Figure 1B.

Angiogenesis modeling starts with the entrapment of tissue spheroids in hydrogels containing ECM proteins (mostly, collagen type I [36,90,91,92] or fibrin [37,81], or, less often, Matrigel [65]). After one to two days of culture, several parameters of sprouts are analyzed. To describe the sprouting process quantitatively, the cumulative sprout length parameter (CSL), defined as total distance from the center of the spheroid to the tip of each sprout of the spheroid (sometimes of the three or five longest sprouts), is commonly used [84]. In addition to CSL, characteristics such as average sprout length per spheroid, average number of sprouts per spheroid, sprouting area, number of branching points, and mean sprout diameter are also applied to analyze the sprouting process and compare the experimental groups. Usually a combination of parameters is used. To evaluate the endothelial-stromal cell interactions during sprouting, the EC and mural cell sprout coverage is analyzed as a percentage of total sprout length. With regard to the software, the most popular as of 18 November, 2021 was the free ImageJ image processing program (https://imagej.nih.gov/ij/ (accessed on 18 November 2021)). In addition to basic software functions, plugin Angiogenesis Analyzer designed for the fibrin bead assay [93] can be installed. 

**Figure 3:**
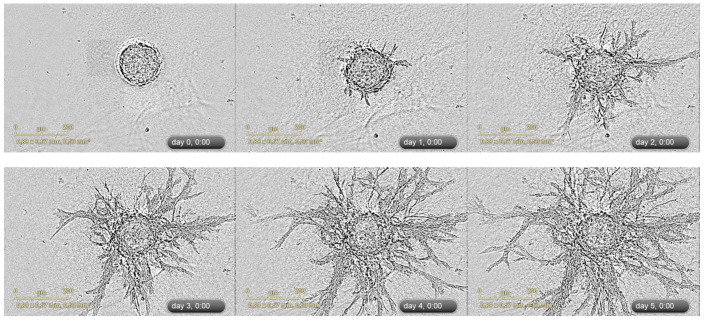
An example of 3D spheroid sprouting assay. Representative images of sprouting of heterotypic HUVEC-UCMSC 3D spheroids were acquired with IncuCyte Zoom imaging system (Sartorius, Bohemia, NY, USA). Corresponding video file S1 is available in the Supplementary Materials. The suspension of cells containing HUVECs and umbilical cord MSCs (cell ratio 1:1) was added (100 µL per well) to the non-adhesive U-bottom 96-well plate (Corning, Waltham, MA, USA). After 72 h, the spheroids were collected and embedded in fibrin gel (4 mg/mL) that was supplemented with 20% platelet lysate (PL) and maintained at +37 °C in CO_2_-incubator for 5 days. Phase-contrast microscopy (the scale bar corresponds to 200 µm).

Despite the fact that a 3D spheroid sprouting model has been applied for more than 20 years, it is still unclear what should be defined as “sprouts” in case of in vitro modeling of angiogenesis. In the literature, authors describe sprouting very differently, from “radial outgrowth of cells” [82], i.e., migration, to “columns of migrating cells” [75], “multiple contiguous cords” [75], and ‘linear alignment of cells” [80] indicating the formation of specific patterns of cell migration and organization. It raises an important question—should any migration of ECs be considered as sprouting? It is worth mentioning that stromal cells also migrate out from the stromal spheroids and form structures with sprout-like morphology [64,74,82,94]. This phenomenon can be attributed to the pro-migratory properties of collagen and fibrin which maintain cellular adhesion and migration without additional stimulation [95]. This fact indicates that the outbound growth of the cells itself cannot be a measure of proangiogenic effect. However, it likely affects the outgrowth of EC sprouts. The migration of cells is an important step of sprouting, but per se, it cannot ensure the formation of mature organized tubular structures. 3D spheroids-based sprouting angiogenesis is a complex dynamic process which can be described as a type of collective endothelial and perivascular cells migration, following or accompanying the formation of ordered lumenalized structures with smooth and compact cell morphology inside the spheroids. This is schematically presented in Figure 1B. Figure 2 gives a real-life example of the formation of microvessel-like structures within spheroids, while Figure 3 illustrates the outbound angiogenic sprouting.

Despite data interpretation challenges, 3D heterogeneous spheroid sprouting assay is being actively applied to evaluate the effect of mural cells on ECs behavior. To analyze the distribution of cells and sprouts in hydrogels, ECs only, or both ECs and mural cells can be labeled with cell tracker dyes (for instance, PKH26 and PKH67) or transfected with genetic constructs expressing fluorescent proteins (GFP or RFP) to perform live imaging. Though it has been proven that the presence of pericytes is crucial for the formation of mature, stabilized capillaries, data concerning the participation of perivascular cells in sprouting angiogenesis and the mechanisms thereof are controversial. Thus, the ECs coculture with VSMCs or fibroblasts decrease ECs’ sprouting [16,80] and this effect is mediated by direct cell-cell interactions, while paracrine regulation itself is not sufficient to drive this process [80]. Comparing the effect of MSCs, fibroblasts, and placental pericytes, it was reported that pericytes promote the formation of sprouts with smooth and compact morphology and follow these structures while MSCs and fibroblasts migrate from ECs and stay segregated from sprouts [92]. 

Interestingly, it was recently shown that the regulation of ECs sprouting activity by pericytes has temporal and spatial constituents [75]. Pericytes from human placenta initially induce paracrine stimulation of sprouting via the production of HGF. HUVECs over the first eight hours sprout independently of pericytes, and after that the pericytes are recruited to the newly formed sprouts. PDGFR-β signaling promotes the recruitment of pericytes as the knock-down of PDGFR-β in pericytes by small interfering RNA (siRNA) leads to a decrease in EC-pericyte association and an increase in ECs sprouting. By 24 h, essentially all ECs sprouts are followed by pericytes and a further increase of the CSL terminates. In contrast, ECs monoculture spheroids continue to increase both the CSL and the number of sprouts. The direct EC-pericyte contact leads to the inhibition of the stimulatory effect on the ECs sprouting. In the light of the above, the influence of perivascular cells should be analyzed over time. 

In addition to ECs sprouting and migration regulation, perivascular cells (such as fibroblasts and VSMCs) improve ECs viability [16,80]. It was shown that EC monoculture spheroids demonstrated migration of cells into collagen up to 24–48 h [64], but later their viability dramatically decreased and they, therefore, could not form vessel-like structures in a sustained way [75,91]. The results of several studies demonstrated an increased viability of ECs that were cocultured with perivascular cells within spheroids in collagen for up to four to seven days [36,84,90,91]. The perivascular cells can also modulate ECs sensitiveness to proangiogenic growth factors such as VEGF and basic FGF. ECs migrate out of the monoculture spheroids in response to VEGF in a dose-dependent manner [16,64]. However, prolonged co-culture of some ECs with perivascular cells (VSMCs, pericytes, bone marrow-derived MSCs, or osteoblasts) leads to non-responsiveness to VEGF and bFGF stimulation [16,35,75,82]. At the same time, it was shown that sprouting of liver sinusoidal ECs in co-culture spheroids increases in response to VEGF and bFGF [84]. Thus, ECs might have different sensitiveness to bFGF and VEGF depending on their tissue origin.

## 7. Role of ECM in 3D Spheroid Sprouting Model

Due to the 3D nature of this assay, hydrogels as 3D matrix play a key role in the regulation of cellular adhesion, migration, and invasion. In angiogenic models, fibrin and type I collagen are usually used as they are two major components of ECM tissue microenvironment. Historically, collagen type I is widely used in 3D spheroid sprouting assays as it was designed by Korff et al. [15] while fibrin is traditionally used in fibrin bead assays. However, it should be noted that they differentially regulate sprouting angiogenesis. In the adult, fibrin deposition is associated with wound healing, enhancing neovascularization. In contrast, type I collagen is a major component of normal dermis which has minimal angiogenic activities. It is known that during the early stages of wound healing, the wounds are mainly composed of a fibrin-rich provisional matrix that is filled with newly formed vessels. These blood vessels are immature as they are stained weakly for laminin. After a week, as collagen starts to replace the fibrin, many blood vessels regress, however, some capillaries mature to form stable vessels. Thus, the sprouting angiogenesis mainly occurs in fibrin clots in the early granulation phase [95]. 

It is known that ECM regulates the switch between cellular migration and proliferation which determines the cellular invasion behavior (as individual cells or as multicellular sprouts). To provide sprout formation, the microenvironment balances the cellular proliferation and invasion rates [96]. In the 3D sprouting model of mixed EC-MSC coculture spheroids, it was shown that fibrin (1.0 and 2.5 mg/mL) promoted both baseline MSCs invasion and ECs sprouting under serum-free media conditions. Thus, fibrin itself has proangiogenic properties. On the contrary, the 3D culture of spheroids in collagen usually requires the addition of exogenous growth factors, such as VEGF and bFGF, to promote sprouting [80,97]. 

In addition to collagen and fibrin, the platelet lysate (PL) gel is considered a promising hydrogel due to its biocompatibility and growth factor-rich composition. Initially developed as a xeno-free alternative to FBS for cell culture, at the present time PL is gaining increasing attention as an agent for promoting tissue repair and angiogenesis and a 3D matrix. Due to the presence of plasma fibrinogen, PL can be polymerized by the addition of thrombin and CaCl_2_. Depending on the preparation protocol, the concentration of fibrinogen in PL varies greatly and it is possible to achieve the needed consistency after the polymerization. It was shown that PL gels can continuously maintain biologically-relevant concentrations of PDGF-BB (platelet derived growth factor-BB) above 1 ng/mL for 5–20 days [38,74]. 

We found only two studies evaluating EC-perivascular cell spheroids sprouting in PL gel in the literature. In the first, Robinson et al. [74] cultured HUVEC-MSC mixed spheroids in the 50 % PL hydrogel. At day three, robust outbound cell migration was observed, mainly due to MSCs invasion, but invasion of HUVECs was also happening. HUVECs sprouting in coculture spheroids that were embedded in PL gel was the same as in 1.0 mg/mL fibrin but higher compared to 2.5 mg/mL fibrin. MSCs demonstrated increased invasion in the PL gel compared to fibrin hydrogels [74]. In the recent study of Shanbhag et al. [98], heterotypic spheroids containing HUVECs and gingiva-derived progenitor cells (GPCs) were cultured in pure PL gel or PL gel that was supplemented with different concentrations of exogenous fibrinogen to improve rheological properties. After 72 h, the sprouting was comparable in pure PL gel and PL gel with 1.25 mg/mL fibrinogen, while in PL gel that was supplemented with 2.5 mg/mL, it was substantially enhanced. It is worth mentioning that the higher concentrations of fibrinogen (>2.5 mg/mL) decreased both the viability of HUVECs and sprouting [98].

In our own unpublished work, heterotypic spheroids consisting of HUVECs and human umbilical cord MSCs were cultured in fibrin hydrogel in a medium that was supplemented with 20% donor platelet lysate [69]. Real-time monitoring revealed that PL supported both cell types’ migration and contributed to the formation of sprouts (Figure 3 and Video S1). Based on these results, we are currently developing an assay for studying the migration, proliferation, and differentiation of human endothelial and pericyte-like cells in three-dimensional conditions that are largely similar to the natural tissue.

## 8. The Application of 3D Heterotypic Endothelial-Perivascular Spheroid System 

Table 1 contains information about published studies that were dedicated to the application of 3D heterotypic endothelial-perivascular spheroid-based assays in biomedical research that were found in the PubMed database. The database was searched using word combinations “spheroid sprouting”, “spheroid sprouting AND mesenchymal stem cells”, “spheroid sprouting AND MSC”, “spheroid sprouting AND smooth muscle cells”, and “spheroid sprouting AND SMC”. Only articles that were published in English and describing studies that focused on heterogeneous tissue spheroids containing two (or more) human cell types—endothelial and perivascular cells (pericytes, MSC, VSMC, fibroblasts, and osteoblasts)—in hydrogels were selected. From about 200 articles that were initially found by key words, we selected only 17 articles that met those criteria. Basic information about these 17 articles (first author, year, journal), and a brief account of the enclosed scientific data (source of ECs, source of perivascular cells, type of hydrogel, key findings about ECs-perivascular cells interaction during the sprouting process) are shown in Table 1.

The earliest work listed in Table 1 was published in 2001 [16] and also the latest papers were published in 2021 [46,98]. Several tissue sources were utilized to obtain ECs. The most commonly used ECs were HUVECs (*n* = 10), followed by human ECFCs (*n* = 2). The commonly utilized perivascular cells included bone marrow-derived MSCs (*n* = 5), umbilical artery SMCs (*n* = 2), and placenta-derived pericytes (*n* = 2). A total of 13 publications reported the usage of collagen hydrogel (*n* = 13), while others exploited fibrin (*n* = 2), PL gel (*n* = 1), and fibrin with PL combined (*n* = 1).

The aim of most of the studies was to assess the functional properties of the heterotypic spheroids with some of them focusing on the role of perivascular cells in a sprouting process as well as their influence on ECs. Overall, the studies reveal that the coculture system is more relevant than the EC monoculture spheroid model for studying the fundamental processes of angiogenesis.

Only very few studies were concerned with the practical application of heterotypic co-culture spheroids in angiogenic assays. Although the EC-spheroid sprouting assay has been widely used for the preclinical studies of inhibitory properties of pharmacological agents [64], the influence of perivascular cells on ECs sensitiveness to anti-angiogenic drugs in 3D heterogeneous spheroid sprouting system is, as yet, poorly understood. However, the results of two articles analyzing the effect of vatalanib and bevacizumab (both – inhibitors of VEGF) on ECs monoculture spheroids and ECFC-MSC spheroids sprouting demonstrated that IC50 in mixed spheroids was higher than that in the EC-only spheroids and correlated with in vivo antiangiogenic activity of the studied drugs [34,35]. Thus, the co-culture system was more relevant than EC monoculture spheroid model. 

In addition to examining the key role of perivascular cells in the development of capillaries, a few studies were focused on how mural cells affect ECs behavior in disease states such as angiogenic dysfunction in idiopathic pulmonary arterial hypertension [36] and diabetic vasculopathy [91]. Interestingly, the concept of this model can be applied to the perivascular cells considered as a functionality test, in particular, for analysis of pericyte-like capacities or differentiation to VSMCs [48,84,92]. 

## 9. Conclusions

Sprouting angiogenesis is the common response of live tissues to physiological and pathological angiogenic stimuli and its accurate evaluation is of utmost importance for basic research and practical medicine and pharmacology. Angiogenesis research that utilizes 2D monolayer cell cultures has major limitations due to the inability of 2D test systems to adequately mimic the nutrient and oxygen gradients and other essential parameters of the in vivo microenvironment. Analysis of the published data suggests that angiogenesis models that are based on heterotypic cell spheroids containing ECs that are co-cultured with perivascular (mural) cells or MSCs are more suitable for modeling angiogenesis in vitro. Inside heterotypic spheroids, ECs form cord-like structures which start branching and merging with each other and can get partially lumenized. Upon the transplantation of heterotypic spheroids comprising human cells into immunocompromised mice, these intra-spheroid EC structures can fuse with the recipient’s capillary network and get filled with blood. After the transfer of spheroids to different hydrogels, the outward sprouting into the hydrogels is easily detectable under the microscope. Although in the in vitro spheroid-based assays sprouting angiogenesis remains incomplete, probably because of lack of the blood flow, many aspects of the microvessel formation and functioning can be elucidated. ECs, when placed in an appropriate 3D environment, can enter intercellular and cell-ECM interactions that are necessary for coordinating the complex process of angiogenesis.

By definition, heterotypic spheroids comprise two or more types of cells. Spheroids that are designed and used in sprouting angiogenesis assays contain ECs and mural cells, such as pericytes and VSMCs, or other stromal cells—MSCs or fibroblasts. ECs form the intra-spheroid capillary-like network that is capable of outbound sprouting, while mural cells and MSCs stabilize the spheroid structure, the EC network, and the outbound sprouts. However, they also can form sprout-like structures, sticking out of spheroids and interfering with the assessment of sprouting angiogenesis. In addition to ECs and stromal cells, other relevant cell types, for example cancer cells, can be added and their impact on angiogenesis and growth characteristics in the proangiogenic environment can be studied [100,101].

Specifically, it has been revealed that 3D co-culture of ECs with mural cells enhances EC properties. Compared to mono-culture spheroids, ECs in co-culture spheroids combining ECs and MSCs display better viability that is required for the long-term experimental studies [75,82,89,91]. High biological relevance of EC-containing co-culture spheroids has been further supported by the in vivo transplantation results that demonstrated the formation of dense tubular vessel-like networks [88,89]. Advances in the development of bioprinting methods have allowed EC-containing co-culture spheroids to be successfully used as building blocks for prevascularized tissue grafts [102]. Following in vivo implantation of the bio-printed endothelial network, anastomosis between it and host circulation was observed with the functional donor ECs-lined blood vessels featuring the graft recipient’s red blood cells inside [103]. 

As pointed out by Franchi-Mendes et al. [60], one of the advantages of spheroid assays is the possibility of focused manipulations with cells to assist elucidation of specific hypotheses. One of more cell types can be genetically altered or specifically labeled enhancing the probative value of the obtained results.

Our search of the available literature revealed papers reporting a variety of both endothelial and mural cell sources that were used to produce co-culture spheroids. The presented results indicate the assay’s flexibility allowing its adaption to cells of different origins, which opens new possibilities for creating tissue-specific angiogenesis models that are based on spheroids containing the tissue’s own cells. One of the fields where these technologies might be applied is the treatment of ischemic conditions, including myocardial infarction and ischemic stroke, remaining the ongoing problems of medicine, including regenerative medicine. With regard specifically to brain ischemia, despite a significant progress in the area of engineering of brain vasculature which was made possible due to microfluidic technologies [104], most of the current model systems are designed to analyze vessel wall functions, i.e., the blood-brain barrier [105,106], but not to study vessel growth and remodeling. 3D angiogenic sprouting models that are based on spheroids comprising of cells that constitute the blood-brain barrier or even the whole neuro-vascular unit, namely, brain ECs, pericytes, and astrocytes, may provide new insights into the mechanisms of reparative angiogenesis occurring during post-stroke regeneration. A better understanding of the mechanisms that are involved in this process could create new opportunities for battling brain ischemia-induced life-threating conditions [107]. The same approach can be applied to the heart muscle ischemia research and the studies of other ischemic diseases. And, of course, 3D sprouting angiogenesis models that are based on spheroids can be and should be extensively used in tumor neovascularization research and anticancer drug testing. In this case, cancer fibroblasts can be used as stromal cells and cancer cells should be added to ECs and stromal cells during spheroid formation.

A major obstacle for the wider use of the spheroid-based angiogenesis assays is the lack of standardized experimental protocols that are needed for generating unified and reproducible data. Although a number of guidelines for preparing angiogenic co-culture spheroids in a supportive 3D environment were proposed [108,109], the analysis of the results remains tricky. Different groups of researchers assess sprouting progression by different parameters depending on the lab’s preferences, techniques available, and skills [32]. One possible solution to the above problem would be the development of affordable all-in-one assay kits [110] in conjunction with software for automated quantitative analysis of angiogenic sprouting [111,112]. Since today’s technological progress makes it easy for biotechnology companies to deliver such products, this future is around the corner. On the other hand, the fast development of innovative methods in cell biology and biotechnology provides excellent milieu for overcoming the still-remaining obstacles.

## Figures and Tables

**Table 1:** Publications that are dedicated to the application of 3D heterotypic endothelial-perivascular cell spheroid-based assays in biomedical research. Only articles that were published in English and describing studies that focused on heterogeneous tissue spheroids containing two (or more) human cell types—endothelial and perivascular cells (pericytes, MSC, VSMC, fibroblasts, and osteoblasts)—in hydrogels were selected from the PubMed database.

Author(s)	Year	Type of ECs	Type of Perivascular Cells	Hydrogel	EC-Perivascular Sprouting Regulation in 3D Coculture Spheroid Sprouting Model (Main Findings)	Reference
Korff T. et al.	2001	HUVECs	Umbilical artery SMCs	Collagen (from rat tails)	ECs form layer of cells on the surface of the coculture spheroid. The presence of SMCs decreases EC sensitiveness to VEGF and bFGF stimulation and apoptotic rate in long-term culture.	[16]
Wenger et al.	2004	HUVECs	Osteoblasts	Collagen	Osteoblasts decreased ECs sensitiveness to VEGFR and bFGF stimulation. The sprouts in co-culture spheroids are mainly composed of osteoblasts and do not form lumens comparing to ECs monoculture spheroids.	[82]
Wenger A. et al.	2005	HUVECs	Dermal fibroblasts	Collagen	Coculture with osteoblasts decreased heterotypic spheroid sprouting compared to ECs monoculture spheroids. The inhibitory effect of fibroblasts was not mediated by paracrine regulation.	[80]
Gluzman Z. et al.	2007	Saphenous vein ECs	Saphenous vein SMCs	Collagen	The activation of Ang-1 in ECs and VEGF in SMCs in coculture sprouting model leads to extensive sprouting.	[90]
Witz et al.	2008	HUVECs, liver sinusoidal ECs (LSECs)	Umbilical artery SMCs	Collagen (from rat tails)	Coculture with SMCs inhibited HUVECs sensitiveness to VEGF and bFGF stimulation. In contrast to HUVECs, LSECs sensitiveness was not affected by coculture with SMCs.	[84]
Chang et al.	2013	HUVECs	Placenta-derived pericytes	Collagen	PCs promote endothelial sprouting by elaborating HGF, but when recruited to invest endothelial sprouts by PDGF-BB, limit the extent of sprouting in vitro.	[75]
Blocki et al.	2013	HUVECs	Placenta-derived pericytes/ bone marrow-derived MSCs /fetal lung fibroblast cell line IMR-90	Collagen	Placenta-derived pericytes improved the integrity of sprouts, while MSCs and fibroblasts migrated larger distances away from ECs and, thus, segregating from sprouts.	[92]
Chang-Hwan et al.	2014	ECs from mesenteric and gastroepiploic arteries	SMCs from mesenteric and gastroepiploic arteries	Collagen	Vessel growth was aligned with MSCs expressing PDGFR-β (a pericyte marker). When MSCs were depleted after lumen formation, vascular structures were collapsed.	[91]
Kim et al.	2015	HUVECs	Cord blood-derived MSCs	Collagen	The combination of three angiogenic GFs PDGF+VEGF+FGF increases the length and number of branches comparing to mono VEGF and dual combinations (VEGF+FGF and VEGF+PDGF).	[99]
Robinson et al.	2016	HUVECs	MSCs (undefined)	Fibrin/PL gel	MSCs influence HUVECs sprouting ability decreasing it.	[74]
Bauman et al.	2018	Umbilical cord OECs	Bone marrow-derived MSCs	Fibrin	OEC-MSC coculture spheroids deposit ECM (fibronectin, collagen type IV) and exhibit robust sprouting.	[81]
Shah et al.	2018	Endothelial progenitor cells	Bone marrow-derived MSCs	Collagen	ECs coculture with perivascular cells in spheroids exhibits IC50 of vatalanib (VEGF inhibitor) correlating with in vivo results	[34]
Shah et al.	2019	Human ECFCs	Bone marrow-derived MSCs	Collagen	The presence of perivascular cells in coculture spheroids affects the sensitiveness to bevacizumab (VEGF inhibitor) which results in relevant IC50 in comparison to EC monoculture spheroids.	[35]
Barnes et al.	2019	Pulmonary arterial ECs from healthy donors and patients with idiopathic pulmonary arterial hypertension (IPAH)	Pulmonary arterial SMCs from healthy donors and patients with IPAH	Collagen type I (from rat tails)	ECs and SMCs from patients with IPAH in coculture spheroids exhibit more robust sprouting due to altered glucose uptake and dysregulation in OGT/O-GlcNAc axis.	[36]
Vorwand et al.	2020	Human ECFCs	Bone marrow-derived MSCs	Fibrin	Spatial localization of ECs within EC-MSC co-culture spheroids affects sprouting potential and NOTCH3 expression.	[37]
Zhang et al.	2021	HUVECs	Dental pulp MSCs	Collagen type I	Pre-treatment with TGF-β1 affects angiogenic properties of dental pulp MSCs on coculture spheroid sprouting assay in comparison to untreated MSCs.	[48]
Shanbhag et al.	2021	HUVECs	GPCs	PL gel	Direct coculture with GPCs in mixed spheroids in a 5:1 ratio significantly improves HUVEC sprouting.	[98]

HUVECs = human umbilical vein endothelial cells, SMCs = smooth muscle cells, ECs = endothelial cells, PDGF-B = platelet-derived growth factor B, ECM = extracellular matrix, VEGF = vascular endothelial growth factor, CSL = cumulative sprout length, Ang-1 = angiopoetin-1, MSCs = mesenchymal stromal cells, ECFCs = endothelial colony forming cells, OECs = outgrowth endothelial cells, GPCs = gingiva-derived progenitor cells.

## Data Availability

Not applicable.

## Supplementary Materials

The following is available online at https://www.mdpi.com/article/10.3390/biology11010018/s1, Video S1: 3D Spheroid Sprouting assay.
